# Effects of Tidal Action on Pollination and Reproductive Allocation in an Estuarine Emergent Wetland Plant–*Sagittaria graminea* (Alismataceae)

**DOI:** 10.1371/journal.pone.0078956

**Published:** 2013-11-11

**Authors:** Yanwen Zhang, Lihui Zhang, Xingnan Zhao, Shengjun Huang, Jimin Zhao

**Affiliations:** 1 Department of Biology, Eastern Liaoning University, Dandong, China; 2 Department of Biology, Changchun Normal University, Changchun, China; Institut Mediterrani d’Estudis Avançats (CSIC/UIB), Spain

## Abstract

In estuarine wetlands, the daily periodic tidal activity has a profound effect on plant growth and reproduction. We studied the effects of tidal action on pollination and reproductive allocation of *Sagittaria graminea.* Results showed that the species had very different reproductive allocation in tidal and non-tidal habitats. In the tidal area, seed production was only 9.7% of that in non-tidal habitat, however, plants produced more male flowers and nearly twice the corms compared to those in non-tidal habitat. An experiment showed that the time available for effective pollination determined the pollination rate and pollen deposition in the tidal area. A control experiment suggested that low pollen deposition from low visitation frequency is not the main cause of very low seed sets or seed production in this plant in tidal habitat. The negative effects of tides (water) on pollen germination may surpass the influence of low pollen deposition from low visitation frequency. The length of time from pollen deposition to flower being submerged by water affected pollen germination rate on stigmas; more than three hours is necessary to allow pollen germination and complete fertilization to eliminate the risk of pollen grains being washed away by tidal water.

## Introduction

Most perennial flowering plants combine sexual reproduction with some form of clonal reproduction [1], [2]. Sexual reproduction through seeds is considered advantageous in a heterogeneous environment or with pathogens, and clonal reproduction via bulbils, corms or rhizomes is considered more successful in stable habitats [3], [4]. Many life history traits in plants show phenotypic plasticity in heterogeneous habitats [5], and there may be a trade-off between life history traits of plants. This is the most basic hypothesis of life history evolution theory [6]. For example, an increase in seed production (sexual reproduction) may lead to a decrease in resources available for the development of vegetative structures (asexual reproduction) [7], [8]. In addition, plant resource allocation between sexual and asexual reproduction can change in different environments [9], [10]. For example, there is an interaction between genotype and environment in the resource allocation of sexual reproduction and clonal growth as well as foraging characteristics in *Ranunculus reptans* [11], [12]. This suggests that the plasticity of life history traits in clonal plants may be an adaptative strategy for life in different environments [2], [13].

Although many research results support this hypothesis [14]–[17], not all plants exhibit trade-offs in life history strategy. For example in *Butomus umbelatus* and *Solidago altissima* there was no trade-off between sexual reproduction and clonal growth [1], [18]. So, what factors influence the trade-off between plant life history strategies? It is not difficult to find, based on previous studies, that individual size, density and environmental conditions also may influence a trade-off between reproductive modes [19], [20].

The sexual reproduction of entomophilous plants is often affected by many factors such as temperature, rainfall, pollinator activity and pollen source. Pollination success or failure has also been shown to affect reproductive allocation by influencing fruit set [21], [22]. However, few studies have focused on the plants living in complex habitat, for example, living in tidal habitat, where their growth and reproduction are not only affected by the matrix of conditions and population density, but also by tidal activity. These plants experience extreme water level changes twice a day, and may be completely submerged, including during the flowering season. Thus, tidal action creates changing pollination environments, presenting many flowering plants with unique challenges during their sexual reproduction [23]. However, tidal action can also bring large nutrition and oxygen benefits for root and underground reproductive structures needed for growth, and may also reduce herbivores [23]. Therefore we hypothesize that for plants living in estuarine wetland tidal habitats, tidal action will affect sexual reproduction negatively and affect asexual reproduction positively. As a result, plants living in tidal habitat may invest different resources to sexual or clonal reproduction, reflecting a different life history strategy compared to plants living in non-tidal habitat.

In order to test our hypothesis, *Sagittaria graminea*, a perennial aquatic flowering plant with sexual and clonal reproduction was selected for study. The species usually occurs in tidal habitats of estuarine emergent wetlands or in non-tidal habitats of rice fields and ditches [24]–[26]. We particularly addressed the following questions: 1) how does reproductive allocation differ between both reproductive patterns in the tidal and non-tidal habitats? 2) how does reproductive allocation differ between sexual and asexual reproduction in tidal and non-tidal habitats? 3) how does water affect pollen germination and stigma receptivity? Finally, is there a trade-off relationship between the two reproductive patterns?

## Materials and Methods

### Ethics Statement

- i: specific permission was not required for study at our site;
- ii: endangered or protected species do not grow in the study site.

### Study Species

*Sagittaria graminea* is a perennial aquatic clonal plant native to North America. The species has become invasive in the Yalu River estuary wetland as well as in nearby rice fields and ditches in China [26]. It is monoecious, with unisexual flowers and an apocarpous gynoecium (159±37 carpels per pistillate flower). Propagation of *S. graminea* takes place in two ways, dispersal of seeds and transportation of clonal fragments by tides [26]. Both seedlings and daughter ramets from corms can develop into flowering and fruiting individuals.

In our study area, the flowering season is from July to September. Each individual has 3 to 12 raceme inflorescences, which are about 40–60 cm tall. Each inflorescence has 3 female flowers that bloom for half a day in the basal whorl followed by 3 to 24 male flowers in apical whorls that bloom for 3 to 5 days. The opening time of most flowers is in the morning 5∶00–6∶00 AM [27]. The species is self compatible but insects are necessary for successful pollination and fruiting under natural conditions. Moreover, individuals exhibit protogyny at the inflorescence level. Fruit ripening and seed dispersal occur in approximately 50 days. Individuals produce flowering shoots as well as rhizomes and corms in the Yalu River estuary wetland. This suggests that the local population is maintained by a combination of both sexual reproduction and clonal growth. Perenniality is typically achieved through rhizomatous growth because above-ground shoots tend to deteriorate over the course of a growing season in the study area.

### Study Site and Tidal Action

The study site is located in the Yalu River estuary wetland in eastern Northeast China (40°05′36″/124°21′29″, n/e). It is a typical tidal habitat and is 30 km from the Yellow Sea. Plants inhabiting this region experience tidal inundation twice a day.

Tides are a regular event and each tide is composed of two processes taking place over about 12 hours. When the water level rises from the lowest to the highest level, this is referred to as rising tide. The ebb tide, on the other hand, is when the water level falls from the highest level to the lowest. The duration taken by these two events is similar. Every day the onset of a tide is about 50 minutes earlier than that of the following day. Water levels of each tide are always dependent on the lunar calendar. During each tide, *S. graminea* living on shore will be submerged for 4 to 6 hours, with inflorescences up to 100–400 cm under water, depending on the location they inhabit along the estuary wetland and the tide levels.

### Reproductive Allocation Modes under Tidal and Non-tidal Habitats

In order to explore the differences in reproductive allocation between sexual and asexual production (flower and seed production as an indicator of sexual production and corms as an indicator of asexual production) under tidal and non-tidal habitat, a manipulative experiment was designed. Plant materials used were from a single patch in the population at the end of October 2010 in the Yalu River Estuary wetland in Liaoning Province, China. On detection of initial signs of senescence of the above-ground parts, corms were harvested at the end of the growing season. More than 100 corms of fairly similar size (diameter 8.22±0.55 mm, n = 30) were collected and stored in the dark at 5°C. In early April 2011, 60 corms were cultivated separately in 60 plastic pots (30 cm in diameter and 32 cm in height) containing soil (5 kg/pot) up to a height of 25 cm. Nutrient-rich black soil was selected to culture these plants.

After settling occurred in the pots, these plants were randomly divided into two sets of A and B. One week later, an appropriate experimental site (tidal habitat, S_1_) was selected and 30 pots of set A (A_1_–A_30_) were placed in the site. These were buried and marked with ‘habitat flags’ in a mudflat in order to keep them from being washed away by the tides. Another experimental site (non-tidal habitat as control, S_2_) was selected on the edge of rice fields 300 meters away from S_1_. An additional 30 pots of set B (B_1_–B_30_) were put in S_2_ at regular intervals (approximately 50 cm). The plants in S_1_ were submerged by two tides every day. Plants in S_2_, on the other hand, grew permanently in shallow water and were not subjected to tidal activity.

In mid July, plants in both S_1_ and S_2_ were flowering. Readily available published tide tables were used when visiting the plants once every week at low tide, when number and sex of flowers were recorded throughout the flowering season. In the course of the investigation, fruits that were just about to mature were collected and put in envelopes in case the seeds fell off. All fruits were air-dried in a laboratory and total seed production per individual was counted. When vegetative structures began to wither at the end of September 2011, all pots were taken out and corms were sieved from the soil and carefully counted and measured.

### Effect of Tidal Action on Pollination

To explore the effects of daily tidal action on pollination in *S. graminea*, the duration over which fresh flowers may have been exposed to pollinators was calculated relying on tide tables. The possibility of female flowers being pollinated was also predicted, by using tide tables and results of field observation to provide a temporal model of tide action, flowering and pollinator visits in the study area (S_1_). We defined the duration when flowers (inflorescences) were above the water surface and were not submerged in daytime as ‘open time’. In addition, based on our observations (see results section), pollinators in the tidal habitats were generally present by about 09∶00 AM and disappeared at about 15∶00 PM if the weather was favorable. That is to say, only in this period of time can flowers above the water surface be visited by pollinators. Thus, we defined the time period when flowers were exposed to pollinators as effective ‘pollination time’. The ‘pollination time’ was usually shorter than ‘open time’. Data from preliminary studies were used to categorize pollination times shorter than two hours as low-possibility for pollination, more than four hours as high-possibility, and intermediate times as mid-probability. The times over which fresh flowers were found above the water surface and pollination expectations are presented in Table 1.

**Table 1:** Open time (duration of inflorescences above the water surface), effective time (flowers open to pollinators) and pollination expectation value (possibility of flowers being pollinated) predicted relying on tide tables in the northern region of the Yellow Sea as well as periods over which pollinators visited flowers (from around 9∶00 AM to 15∶00 PM).

Date of lunar calendar	Open time (daytime)	Pollination time (hour)	Pollination expectation value
1/16	8∶30–14∶40	>5	high
2*/17*	9∶20–15∶30	>5	high
3/18	10∶05–16∶15	4–5	high
4/19*	10∶55–17∶05	4–5	high
5*/20	11∶40–17∶50	3–4	middle
6/21*	12∶30–18∶40	2–3	middle
7*/22	13∶20–19∶30	1–2	low
8/23*	14∶05–20∶10	<1	low
9/24	15∶00–21∶05	<1	low
10*/25	3∶20–9∶30,15∶40–21∶55	<1	low
11*/26	4∶10–10∶20,16∶30–22∶35	1–2	low
12/27*	5∶00–11∶00	2–3	middle
13/28*	5∶50–12∶00	3–4	middle
14/29	6∶35–12∶50	3–4	middle
15*/30	7∶25–13∶40	4–5	high

Pollination expectation value was artificially divided into three levels: high (pollination time longer than 4 hours), medium (pollination time in between 2 to 4 hours) and low (pollination time less than 2 hours). Days with an asterisk are sampling days.

A total of 12 sampling days were selected depending on pollination possibility in July and August, 2011 (indicated with an asterisk in Table 1). One female flower was collected from every different individual and in total collected 60 female flowers in 60 different individuals (naturally-occurring plants close to S_1_) before the end of the effective pollination time on each sampling day (tidal habitat). In the S_2_ non-tidal habitat, we only collected one female flower from every different individual and in total collected 30 female flowers, at 17∶00 PM. Sample flowers were examined by pocket lens and the number of pollen grains deposited on a single stigma of pollinated flowers was determined using a dissecting microscope (at least 10 stigmas from each pollinated flower), to determine pollination rates (pollinated flowers/total flowers × 100%). In addition, visitation frequencies of pollinators were also observed in different periods in the two investigated habitats. Observations in the non-tidal habitat were from 07∶00 AM to 17∶00 PM, in tidal habitat were made only in effective ‘pollination time’. Observation periods were 20 minutes each two hours and repeated at least 4 times in the flowering season.

### Effect of Water on Pollen Germination and Stigmatic Receptivity

Based on the fact that actual seed sets of this species in tidal habitat are far lower than pollination rates, it was hypothesized that there were likely other factors such as flooding (tides) that affected pollen deposition or/and germination on the stigma or stigma receptivity. In tidal habitat floating material can impact stigmas, so that it is impossible to examine the pollen lost (e.g. pollen grains were washed away by water) on stigmas and pollen germination during rising tide, thus, in order to validate our hypothesis, a manipulation experiment was designed in 2011.

In an experimental greenhouse, over 60 individuals of *S. graminea* were planted from the study population in plastic pots with 3 plants per pot. When these individuals started flowering, they were covered with cages to prevent visits by pollinators. Experiments were performed from 09∶00 AM to 13∶00 PM. Six numbered large plastic buckets (90 cm in diameter and 100 cm in height) were prepared to ensure that the plants placed in the plastic pots would be completely submerged under water. Hand pollination was then carried out on female flowers that opened on the same day using male flowers from different individuals. After pollination, the pots were divided into six groups, 3 pots per group with a total of 9 plants and over 30 female flowers. These pots were then placed in the large plastic buckets to simulate tidal submersion 0, 1, 2, 3 and 4 hours after hand pollination. Another group of individuals was not placed in a bucket as a control group to examine pollen deposition per stigma and pollen germination rate in natural condition. We also placed 3 un-pollinated individuals into another bucket for 5 hours to determine the effect of water on stigmatic receptivity by the benzidine H_2_O_2_ test [28].

After five hours, plants that had been submerged (un-submerged flowers as control) were removed from the buckets and all the pollinated female flowers picked. These flowers were separately stored in small bottles with fixation liquid (alcohol: acetic acid, 3∶1) in order to monitor pollen tube growth in the pistils. We randomly checked 10 stigmas per flower and in total checked 10 flowers for every set of samples. Their total pollen deposition and the germinated pollen grains per stigma were examined [29], [30] and we calculated pollen germination rate (the germinated pollen grains/total pollen deposition %) as well as pollen loss rates (pollen deposition of control flower - pollen deposition of soaked flowers/pollen deposition of control flower %).

We also tested pollen germination in natural and in cultural conditions. To determine pollen germination on stigmas in natural condition we hand pollinated flowers, then collected stigmas from these flowers by interval sampling, and then counted pollen as described above. In cultural conditions, the mature anther was shaken on a slide in order to let pollen grains fall on the slide, and then a drop of water was placed on the slide and put under room temperature to culture. Pollen germination was examined after 10 min, 20 min, 30 min, 60 min and 120 min under a microscope. This experiment was repeated three times.

### Data Analyses

Procedures used for statistical comparisons between tidal and non-tidal habitat depended on whether there were replicate measurements per plant or not. For variables consisting of single data points per plant (e.g. corms and rhizomes) comparisons between the two habitats were performed using nonparametric Kruskal–Wallis analysis of variance when data departed from normality and Student *t*-tests if data were normally distributed. Generalized linear mixed model were used when true within-plant replicates of count proportion data were available for individual plants (e.g. Number of females or male flowers per inflorescences, fruit set).

Estimates of pollination rate were analyzed by fitting generalized linear models to the data. Rate of pollination was modeled as a binomial process using logits. Regression analysis was used to compare the relationship between the effective ‘pollination times’ and pollination rates or pollen grains per stigma.

A MANOVA was used to test differences in pollen germination rates and pollen loss rates between treated and control flowers as well as between and among submergence time treatments. To fulfill requirements of ANOVA, all data were analyzed for normality of variance prior to analysis. Percentage data (e.g. pollination rates or germination rates) were arcsine transformed. Furthermore, simple t-tests (least significant difference [LSD]) were used to control the Type I error rate in the multiple pair-wise comparisons.

Significance level was set at 0.05. All statistical analyses were carried out in SPSS 16.0 statistical package.

## Results

### Reproductive Allocation Modes under Tidal and Non-tidal Habitat

Results showed that the species had very different reproductive allocation modes in the two different habitats. In tidal habitat, although plants produced a similar number of female flowers compared to those in the non-tidal habitat (*χ^2^* = 0.462, *d.f.* = 1, *P*>0.05), they produced more male flowers (*χ^2^* = 6.414, *d.f.* = 1, *P*<0.01) and total flowers (*χ^2^* = 3.867, *d.f.* = 1, *P*<0.05) compared to those in the non-tidal habitat (Table 2). However, because fruit sets were very low in the tidal habitat, seed production (203.5±154.2 per individual) was only about 9.7% of that in the non-tidal habitat (2087.8±519.4 per individual). The difference was highly significant (*χ^2^* = 135.661, *d.f.* = 1, *P*<0.001). In addition, significant differences were detected in asexual reproductive outputs such as the number of corms (*χ^2^* = 13.245, *d.f.* = 1, *P*<0.01) between the tidal and the non-tidal habitat (Table 2).

**Table 2:** Effects of tidal action on sexual and asexual reproductive output between tidal habitat and non-tidal habitat in *Sagittaria graminea.*

Sources	Number of flowers			Seed production	Corms
	Total	Female	Male		
Tidal habitat	50.5±6.1^a^	13.7±2.3^a^	36.8±4.8^a^	203.5±154.2^a^	36.9±7.2^a^
Non-tidal habitat	43.5±4.4^b^	15.1±2.2^a^	28.3±4.4^b^	2087.8±519.4^b^	20.3±5.8^b^

Sample sizes were 30 individuals in each treatment. The same letters do not differ significantly at *P*<0.05 (Mean ±1sd.).

### Effect of Tidal Action on Pollination

**Figure 1:**
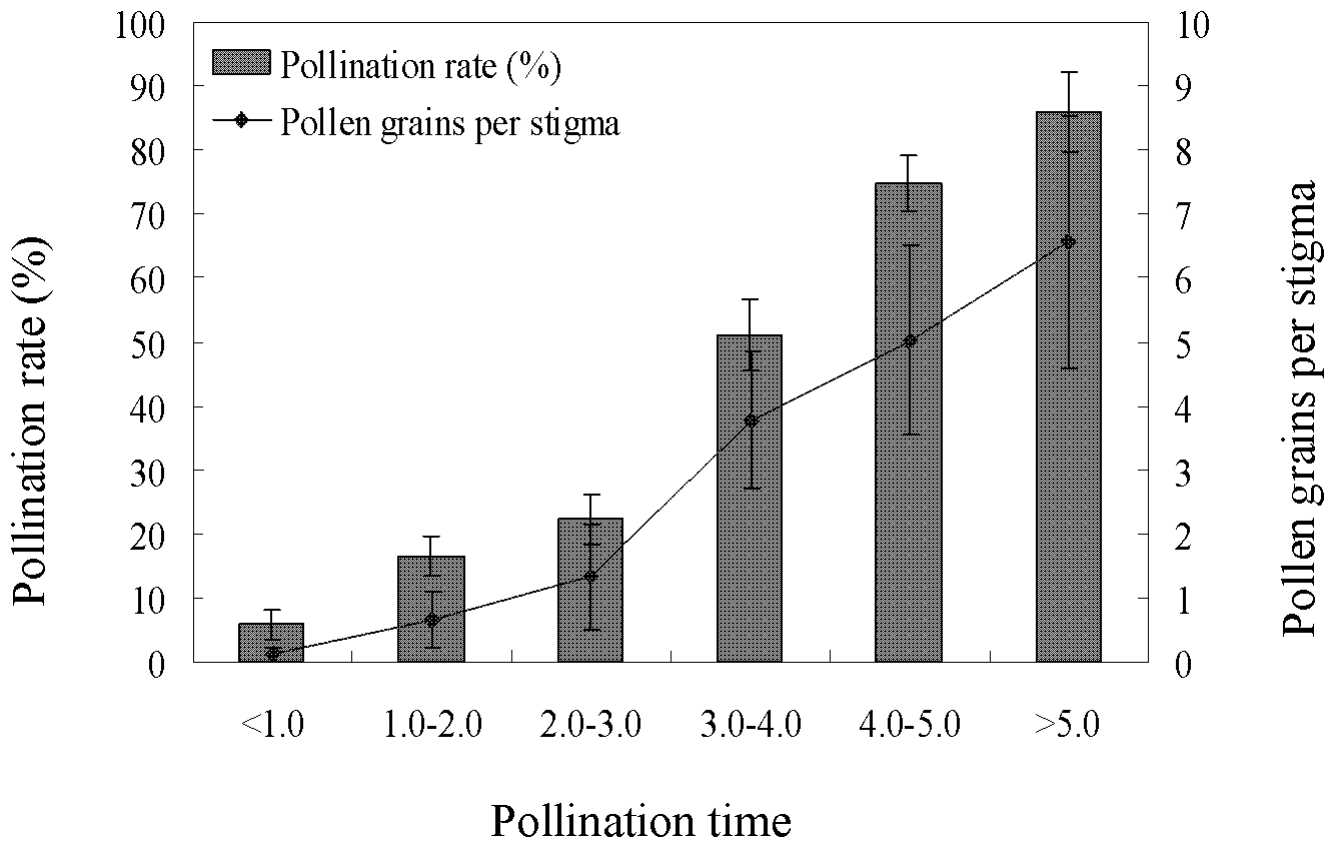
Effects of durations over which flowers were open to pollinators (pollination time) on pollination rate and pollen deposition. Pollination times were calculated from tide tables. (Mean ±1sd.).

Results suggested that in tidal habitat, when the effective ‘pollination time’ of these flowers was less than 2 hours, pollination rate and pollen deposition were very low. When the effective ‘pollination times’ were longer than 3 hours, pollination rate and pollen deposition increased considerably. A majority of flowers was pollinated when the effective ‘pollination times’ were longer than 5 hours (Fig. 1). There was a significant positive relationship between the effective ‘pollination times’ and pollination rates of flowers (*Y = 17.291×* − *17.787*, *r^2^* = 0.9605, *P*<0.001) as well as number of pollen grains per stigma (*Y = 1.3669x* − *1.8707*, *r^2^* = 0.9606, *P*<0.001). In non-tidal habitat, nearly 100% of flowers were pollinated, with a mean of 13.6 (±9.2, n = 120) pollen grains deposited on each stigma. Even those flowers in tidal habitat with effective ‘pollination times’ longer than 5 hours had a mean pollen deposition of only 6.6 (±4.7, n = 120), significantly less than in non-tidal habitat (*t*-test, *t* = 65.715, *P*<0.001).

**Figure 2:**
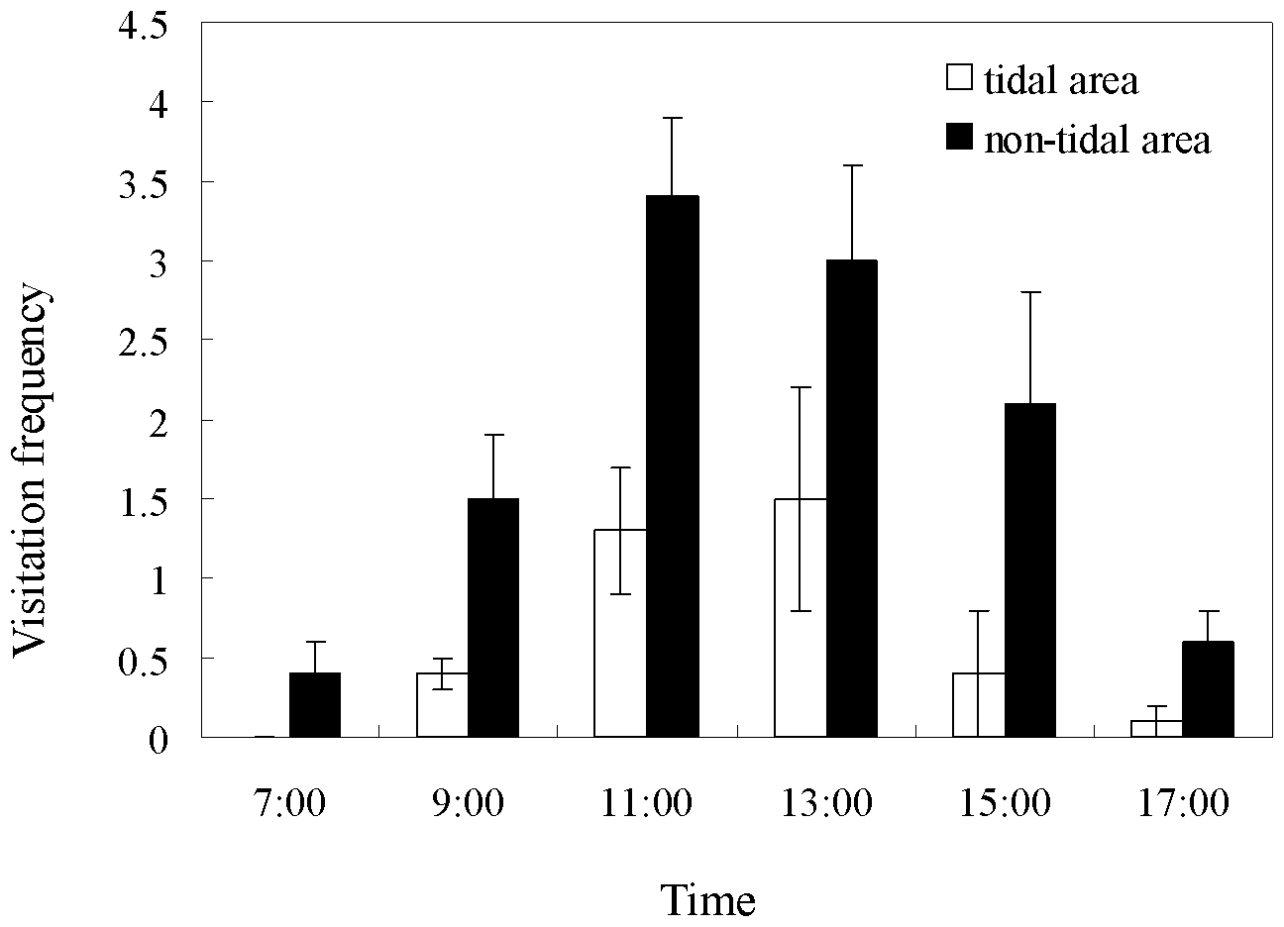
Frequencies of pollinators visiting *Sagittaria graminea* individuals in the tidal and non-tidal area at different times. Frequencies are times pollinators visited a single inflorescence in a 20-minute observation period from 7∶00 AM to 17∶00 PM (Mean ± sd.).

*Apis cerana*, *Halictus sp*. and Syrphidae (*Sphaerophoria sp*. and *Eristalls sp*.) were the most frequent flower visitors observed in both habitats. Bee visit frequencies were much higher than for the flies in the study populations and bees were the most effective pollinators of *S. graminea*. The male and female flowers of *S. graminea* secrete nectar so that there was no obvious preference for bees visiting flowers. In the tidal habitat, pollinator activity took place from around 09∶00 AM to 15∶00 PM (if this period is not subject to tidal activity), while in the non-tidal habitat, it was from around 08∶00 AM to 17∶00 PM, with an almost doubled visit frequency compared to tidal habitat (Fig. 2). Based on results from each observation period, in the tidal habitat plants received 15.3 (±3.7, n = 4) visits on each inflorescence on a fine day (about six hours of pollination time), so that the pollination time of one hour per inflorescence would result in 2.5 visits. While in the non-tidal habitat, plants received 67.5 (±9.1, n = 4) visits on each inflorescence on a fine day (about nine hours pollination time), a mean of 7.5 visits per hour per inflorescence. A significant difference was detected in visitation frequencies between habitats (*t*-test, *t* = 122.657, *P*<0.001).

### Effect of Water on Pollen Germination and Stigmatic Receptivity

**Figure 3:**
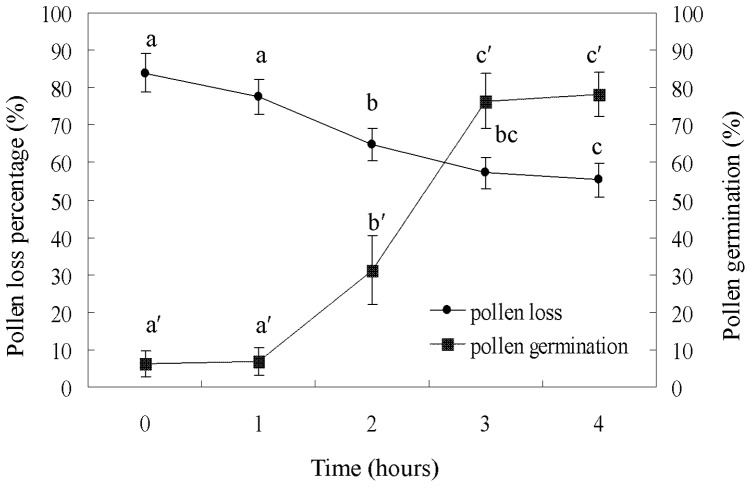
Effects of the duration from flower being pollinated to flower being submerged by water (waiting time) on pollen loss rates and pollen germination rates in *Sagittaria graminea*. The same letters do not differ significantly at *P*<0.05 (Mean ±1sd.).

Test results showed that after uniform hand pollination of flowers, primary pollen deposition averaged 21.7 (±9.2, n = 100) grains per stigma. The duration from flower being pollinated to flower being submerged by water affected the pollen loss, e.g. if flowers were inundated by water just after they were pollinated, their effective pollen deposition was reduced to 3.5 (±2.7, n = 200) pollen grains per stigma, and about 83.9 (±9.1)% of pollen grains were washed away by water. If the duration exceeded two hours, the effective pollen deposition was 7.6 (±5.4, n = 100), and pollen loss was 64.9 (±4.4)%, and if the duration exceed four hours, then the effective pollen deposition was 9.7 (±5.8, n = 100), and pollen loss was 55.3 (±4.6)%. In all five experiments, effective pollen deposition was significantly less than primary pollen deposition (MANOVA - test, *df* = 5, *P*<0.01) (Fig. 3).

In addition to the pollen loss from submergence in water, the length of time from flower being pollinated to flower being submerged also affected germination of the deposited pollen. If flowers were inundated by water just after they were pollinated, pollen germination was very low (6.2±3.5%, n = 100). When the length of time exceeded two hours, pollen germination increased to 31.3 (±9.1)%; the difference was significant (MANOVA - test, *F_1,198_* = 35.264, *P*<0.001). When the length of time exceeded three hours, germination increased to 76.4 (±7.3)%, which was not significantly different from the results of the control (79.2±5.8%) (*F_1,198_* = 1.665, *P*>0.05) (Fig. 3).

In addition, regression analysis showed that there was a significant negative relationship between the length of time from flower being pollinated to flower being submerged and pollen loss (*Y = −7.75x* +*83.26*, *r^2^* = 0.9529, *P*<0.001) as well as a significant positive relationship between the length of time from flower being pollinated to flower being submerged and pollen germination (*Y = 21.35x − 2.9*, *r^2^* = 0.8942, *P*<0.001).

Although stigmas were still receptive after 5 hours of inundation by water, pollen germination experiments showed that in natural conditions (temperature 25–30°C) pollen germination on a stigma required at least 30 minutes. Pollen could not germinate in the water and grains would begin to burst after 20 minutes of soaking. After 30 minutes, about one third of pollen grains would burst and when the duration exceeded an hour, a majority of pollen grains would burst and lose activity.

## Discussion

There may be a linkage between life history traits of clonal plant and environmental features. When clonal plants live in heterogeneous habitats with different levels of resources, genets that obtain resources through changes in their clonal architecture may be favored [31]–[33].

### Reproductive Allocation under Tidal and Non-tidal Habitats

In estuarine wetlands, the daily periodic tidal activity has a profound effect on plant growth and reproduction [34]. The environmental selective pressure on *S. graminea* living in the tidal habitat will inevitably drive the population to increase its cloning ability. Clonality in aquatic environments may be a response to the uncertainty of pollination or a means to exploit stable environments [34]–[37].

Our study results clearly demonstrate that in both habitats, individual modes of resource allocation for reproductive investment were different. In the non-tidal habitat, individuals invested more resources in production of a large amount of seeds so that resources invested in asexual reproduction were relatively reduced. In tidal habitat, sexual reproduction is likely to be affected negatively by tidal action so that plants will invest more resources in production of more corms and rhizomes. In general, there was no significant difference in female flower production between tidal and non-tidal habitats, suggesting similar reproductive efforts in this measure of female fitness. However, poor pollination environment, and more importantly the negative effects of tides, limited extremely the realized female reproductive success. On the contrary, the fact that individuals in the tidal habitat produced significantly more male flowers indicated more reproductive efforts in the context of male fitness, which may be also an adaptive phenomenon. In total, plants have higher reproductive efforts but lower female reproductive outputs in tidal habitat.

Several previous studies have showed that the trade-offs between modes of resource allocation lead to varied conclusions in different species. For example, several studies detected a negative trade-off relationship between reproductive modes [2], [37]–[39], while others do not exhibit a clear trade-off relationship [20], [39]–[42]. Our results support the hypothesis of negative correlation between the two reproductive modes. The variation and trade-off of resource allocation between both reproductive modes show that the species has the ability to adjust its reproductive strategies.

The plasticity of a plant’s reproductive strategies might be a selective advantage so that the plant can effectively adapt to a new habitat [43]. When *S. graminea* occurs in a new habitat as an alien species, individuals may display plastic reproductive strategies. This ability to adjust resource investment between the reproductive modes could be particularly important for alien species in adapting to new heterogeneous habitats and in gaining an advantage over native species in the process of competition.

Previous investigations have shown that the amount of corms is a key factor in determining the population density of the next growing season in the wild where seedlings are relatively rare [44]. Higher ramet densities in a patch are beneficial to colonization and enhancement of competitiveness over adjacent species. Therefore, *Sagittaria graminea* living in estuarine wetlands (tidal habitat) often create monodominant patches even more than 1000 square meters area [44]. However, in non-tidal habitat, large numbers of seeds were dispersed into adjacent area increasing the likelihood of founders in homogeneous or heterogeneous habitats, contributing to the genetic diversity of the population. This phenomenon has been confirmed in other studies [37], [45]. This adaptive strategy allows the alien species to obtain maximum gains from the prevailing environmental conditions and to gain an advantage where they are in competition with native species [46], [47].

An interesting phenomenon was uncovered in the tidal habitat. Individuals there had more flowers than those in the non-tidal habitat. The difference mainly arose in the number of male flowers and not female flowers. Based on the sequential resource adjustment hypothesis [48], individuals in the early flowering season with fewer flowers due to factors such as low pollination levels that led to low fruit production may be compensated by increased numbers in the late flowering season. In monoecious plants, such as *S. graminea*, increasing the number of male flowers that require fewer resources is a more economical way to realize male fitness. A similar situation has been witnessed in other studies [19], [42], [49], [50].

### Effect of Tidal Action on Pollination and Pollen Germination

Observations of visit frequencies of pollinators and pollen deposition showed that if the effective pollination time was more than three hours, most flowers could be pollinated and most stigmas in each flower would receive pollen grains. According to the Table 1, over 50% of days in the flowering season have effective pollination times longer than three hours, hence, most of the flowers can potentially be pollinated. In addition, another study in genus *Sagittaria* showed that the rates of pollen germination on the stigma were 100% and no pollen selection occurred on the stigma [17]. This is similar to our result in *S. graminea.* As shown in Table 1 and Figure 1, less than 30% of flowers endured pollen limitation through lower pollination rates and pollen loads. However, in this genus, a pollen tube reallocation phenomenon can occur in the process of fertilization [29], [30], [51]. Therefore, we believe that pollen limitation is not the main cause of very low seed sets or seed production in this plant in the tidal habitat. The manipulation experiment showed that at least 50% of the ungerminated pollen grains on stigmas would be washed away and pollen grains that remained on stigmas did not germinate in water. Thus the flowers need to remain above water for some period after pollination; based on our results, at least two hours is needed for pollen germination to reach 31.3%. And at least three hours is needed in order to ensure pollen grains are not washed away by water and for pollen tube growth to complete fertilization. According to a recent survey water can significantly interfere with pollen germination in many plants [52]. On the other hand, even if the effective pollination time of some flowers reached 4 to 5 hours with a higher pollination rate, effective pollination was more likely to occur in the later period of effective pollination time. Shorter times would result in some of the pollen on the stigma failing to germinate due to the osmotic pressure of water when flowers were submerged by the subsequent arrival of the tide. Thus, the destructive effects of tides (water) on pollen germination may surpass the influence of low pollen deposition by low visitation frequency.

Conclusions presented here suggest that tidal events may play an important ecological role in influencing resource allocation and pollination and probably an evolutionary role in adjusting reproductive strategy in the study species.
